# Clinical Cases

**Published:** 1889-06

**Authors:** Clarence N. Payne

**Affiliations:** Port Jervis, N. Y.


### CLINICAL CASES.

Clarence N. Payne, M. D., Port Jervis, N. Y.

Case (1).—Patrick M., age thirty-two, coal-heaver. First came to me Feb. 22d, 1888, with the following symptoms of many years duration, which had led him to apply to "every doctor in town:"

Symptoms always aggravated in winter. A gnawing sensation, aggravated, about half an hour after eating, or a feeling as if had not eaten anything. Is especially aggravated by potatoes, cabbage, and beans. Tasteless eructations sometimes relieve. No thirst; fond of meat and fat food.

Alumina$^{200}$ relieved him promptly for that year.

In April this year, patient returned to me with same symptoms, and was again promptly relieved by two doses of Alumina$^{200}$.

Case (2).—Mrs. H., age sixty-five, Dec. 19th, 1888. Pain in left thigh, nature of sciatica, aggravated at night. Cannot stay in bed; has to get up and move about, which relieves; also relieved by heat, and wrapping limb up in flannel. Limb very sore and cold, no sweat, neck stiff, aggravated before storm.

Promptly and decidedly relieved by Rhus.

Case (3).—Baby K., age two and a half months. Dec., 1888. Constant crying since birth, but only during day; sleeps well at night.

Cham.$^{3}$ seemed to aggravate. Bell.$^{30}$, relieved very little. Cham.$^{200}$ relieved entirely and at once all irritableness, so that "baby is as good as can be."

Case (4).—Mrs. Diantha B., aged seventy. Nov., 1888. Neuralgia of many years duration.

Pain in right lower jaw, and some pain above right eye. Of late has been getting much worse. Pain is also felt in side of tongue, and is aggravated by moving tongue. Jaw is sore. Pain is relieved by heat, and aggravated by cold air and cold weather. Cured by Mag. phos.$^{30}$, after the failure of many other remedies to give relief.

